# Unequal on-site interaction effects in the one-dimensional electron system at quarter filling

**DOI:** 10.1038/s41598-021-90336-x

**Published:** 2021-05-24

**Authors:** Hanqin Ding, Yan Xu, Weiting Chen

**Affiliations:** 1 School of Physical Science and Technology, Xinjiang University, Urumqi, 830046 China; 2 Center for Theoretical Physics, Xinjiang University, Urumqi, 830046 China

**Keywords:** Materials science, Physics

## Abstract

The one-dimensional antiferromagnetic correlated electron system described by the unusual *t*–*U*–*J* model with alternating on-site interactions at odd ($U_o$) and even ($U_e$) sites is studied analytically. At weak coupling, the use of bosonization and renormalization-group techniques helps to obtain ground-state phase diagram. At quarter filling, the unequal on-site repulsion ($U_e\ne U_o$) causes the occurrence of umklapp processes and the generation of a charge excitation gap. Contrary to the usual case ($U_e=U_o$), the system is not metallic but insulating. For $U_e+U_o<2J$, the system is in a spin-gapped phase with charge-density-wave (CDW) instability; for $U_e+U_o\ge 2J$, the system is in a spin-gapless phase characterized by the coexistence of both CDW and spin-density-wave (SDW) instabilities, where the SDW correlation dominates over the CDW one.

## Introduction

The discovery of high-$T_c$ cuprate superconductors^1^ has increased the interest in low-dimensional correlated systems, and particularly in the electron system characterized by interactions between the charge and spin dynamics. It is generally recognized that the crucial physics of the novel superconductor is closely related to two-dimensional *CuO* planes^2^. By appropriately doping, the cuprates exhibit a variety of phenomena in their magnetic insulator, metal and superconductor^3^. The 2D *t*-*U*-*J* model is a suitable model for studying a series of phase transitions in the cuprate systems. The RVB spin-liquid state^4^ and the gossamer superconducting state^5^ are proposed as necessary precursors to high-temperature superconductivity at higher doping. In two dimensions, however, it remains very challenging to solve this model exactly. By comparison, more attention has been devoted to the one-dimensional (1D) counterpart^6–10^. Further more, 2D systems share some common properties with the 1D case. To a certain extent, the study of 1D models may act as a good guide to understanding higher-dimensional systems. It is helpful to study the 1D physics as a starting point and then to try to apply it to higher-dimensional cases. After all, 1D models are much easier to handle than their counterparts in higher dimensions and can even be exactly solvable. In one dimension, some analytical techniques, such as bosonization and renormalization-group analysis^11–14^, are effectively used to give the correct physics of models.

The 1D *t*-*U*-*J* model, which incorporates an nearest-neighbor spin exchange, is an extension of the standard Hubbard model. As is known, the Hubbard model^15^ is the archetype of correlated electron systems. In spite of its apparent simplicity, the model does exhibit that the on-site Coulomb interaction has a strong impact on the charge dynamics, leading the 1D electronic system into a Mott insulator at half filling. Away from the half-filled density, however, the system becomes of a conductor. Note that the *t*-*U*-*J* model is not an extension of the *t*-*J* model. Really, the *t*-*J* model is viewed as a descendant of the Hubbard model in the strong-coupling limit ($U/t\rightarrow \infty$), where the double occupancy is completely forbidden. The *t*–*U*–*J* model is by no means the sum of the *t*-*J* and Hubbard models, because it allows for a more flexible arrangement between finite *U* and *J* than reflected in both models. In the case of positive interactions ($U,J>0$), the ground state of the 1D *t*–*U*–*J* model exhibits insulating phases at half filling^6–8^ while is in metallic phases out of half filling^9,10^. The occurrence of such phenomenon is directly connected with the so-called umklapp processes, which originate from the periodicity of lattice model and stand for large momentum transfers. The lattice structure spoils the completely translational invariance, reducing the momentum conservation down to a weaker condition, where the momenta need only to be conserved modulo a vector of the reciprocal lattice (**G**). In one dimension, the Fermi surface is reduced to two symmetric points, and $G=2\pi /a$, with *a* the lattice spacing. The umklapp processes correspond exactly to a momentum transfer of one reciprocal vector, which is equal to four times Fermi momentum ($k_F$) from the lattice. $k_F$ is determined by the electron density $\nu$, defined as $k_F=\nu \pi /2a$. Here, the umklapp scattering refers only to the first order process.

Notice that the above-mentioned result depends on the assumption about the umklapp processes that whether in the Hubbard model itself or in its extensions, all lattices are completely equivalent, characterized by the equal on-site interaction at each site. Within this hypothesis, one gets an umklapp process where two electrons are scattered from one side of the Fermi point to the other only if $4k_F=G=2\pi /a$. The occurrence corresponds to $\nu =1$, which is just the half-filled case. Deviation from such a commensurate filling, the umklapp process does not take place in one dimesion^11,16^. In this case, it is natural to ask the question that, what happens if the assumption is released. That is to say, if the 1D lattices are not equivalent, accompanied by unequal on-site interactions on different lattices, whatever will become of the fate of umklapp processes away from half filling? Really, in the early 1990s, Emery reported that there are different on-site interaction between copper and oxygen sites in the Cu-O chain of undoped $La_2CuO_4$ system^17^. Nevertheless, the less generic situation where the site equivalence of the system is destroyed due to the introduce of several types of lattices, has been intensively discussed. By settling this subject, we will here investigate a 1D electron system with unequivalent lattices at odd and even sites, described by an unusual 1D extended Hubbard model with alternating on-site repulsive interaction at odd and even sites. From the viewpoint of theoretical investigation, our consideration is very essential to understand complex interactions. In one dimension, the model Hamiltonian is given by

1 $$\begin{aligned} H&=  {} -t\sum _{j\alpha }\left( c^\dag _{j+1\alpha }c_{j\alpha }+h.c.\right) +U_o\sum _{j\in odd}n_{j\uparrow }n_{j\downarrow }\nonumber \\&+U_e\sum _{j\in even}n_{j\uparrow }n_{j\downarrow }+J\sum _{j}{} \mathbf{S} _j\mathbf{S} _{j+1}. \end{aligned}$$

As usual, the $c_{j\alpha }^\dag$ ($c_{j\alpha }$) is the creation (annihilation) operator of an electron with spin $\alpha$ ($\uparrow$ or $\downarrow$) on lattice site *j*, and the number operator $n_{j\alpha }=c_{j\alpha }^\dag c_{j\alpha }$. $\mathbf{S} _j$ is the spin operator on site *j* with $S_j^\beta =\frac{1}{2}c^\dag _{j\alpha }\tau _{\alpha \alpha ^\prime }^\beta c_{j\alpha ^\prime }$, where $\tau ^\beta$ are the Pauli matrices ($\beta =x,y,z$). *t* represents a single-electron nearest-neighbor hopping and *J* denotes inter-site exchange interaction. For a change, $U_o$ and $U_e$ denote on-site interaction at odd and even sites, respectively. We only consider the case for repulsive $U_o\ne U_e$ and isotropic antiferromagnetic exchange, $U_o,U_e,J>0$. To highlight the physics of non-half-filled systems, we focus on the quarter filling where the umklapp processes are absent in the usual *t*–*U*–*J* model ($U_o=U_e$)^9^. To use the low-energy field theory technique effectively, we concentrate our attention on the weak-coupling regime, $U_o,U_e, J\ll t$, as in other work^6–10^. The result shows that the unequal on-site repulsions at nonequivalent odd and even sites gives rise to the occurrence of umklapp processes at quarter filling. The charge excitation spectrum is always massive, and the system is not metallic but insulating deviating from the $U_o=U_e$ line. In the spin sector, there is a transition from gapped excitation to gapless one. The ground state phase diagram consists of two kinds of insulating phases. The spin-gapped phase is the charge density wave (CDW). The spin-gapless phase is characterized by the coexisting CDW and spin density wave (SDW) instabilities, however, the SDW correlation is logarithmically dominant with respect to the CDW correlation. Theoretically, the result provides the further understanding of quantum properties of 1D correlated electron systems and the occurrence of umklapp processes.

## Boson representation

Bosonization^18,19^ is a powerful tool to analyze 1D fermion systems and is extensively used to investigate ground-state phases of 1D extended Hubbard models^20–28^. This approach decouples the charge and spin degrees of freedom, and some of the interacting terms in the fermionic Hamiltonian turn into free noninteracting terms in the corresponding bosonic expressions. The 1D low-energy physics is mainly described by these states close to left- and right-Fermi points, around which the fermionic operators are decomposed in the continuum limit as

2 $$\begin{aligned} c_{j\alpha }=e^{-ik_Fx}\psi _{L\alpha }(x)+e^{ik_Fx}\psi _{R\alpha }(x). \end{aligned}$$

Here the fermion fields $\psi _{L,\alpha }(x)$ and $\psi _{R,\alpha }(x)$ describe left-moving and right-moving particles respectively, and they contain only long wavelength components which are smooth within about one lattice spacing.

In order to carry out bosonozation technique, one first needs to introduce density operators by^28^

3 $$\begin{aligned} \rho _{r\alpha }(x)=\psi _{r\alpha }^\dag (x)\psi _{r\alpha }(x), \end{aligned}$$

where $r=L,R$ is the charity index (left or right). Accordingly, the charge- ($\rho _r$) and spin- ($\sigma _r$) density operators are defined as

4 $$\begin{aligned} \rho _r(x)=\frac{\rho _{r\uparrow }(x)+\rho _{r\downarrow }(x)}{\sqrt{2}},\quad \sigma _r(x)=\frac{\rho _{r\uparrow }(x)-\rho _{r\downarrow }(x)}{\sqrt{2}}. \end{aligned}$$

In the case of non-half filling ($\nu \ne 1$), the complete Hamiltonian Eq. (1) is rewritten as (in what follows, we set $a=1$)

5 $$\begin{aligned} H&=  {} \pi v_F\int \mathrm {d}x\left( \rho _L^2+\rho _R^2+\sigma _L^2+\sigma _R^2 -g_\rho \rho _L\rho _R-g_\sigma \sigma _L\sigma _R\right) \nonumber \\&+\frac{\pi v_F}{2}\int \mathrm {d}x\left\{ g_{1\perp }\left( \psi _{R,\alpha }^\dag \psi _{L,\alpha }\psi _{L,-\alpha }^\dag \psi _{R,-\alpha }+h.c.\right) \nonumber \right. \\&\left. +g_{3\perp }\left[ \psi _{R,\alpha }^\dag \psi _{L,\alpha }\psi _{R,-\alpha }^\dag \psi _{L,-\alpha }e^{i\left( \frac{1}{2}-\nu \right) Gx}+h.c.\right] \right\} , \end{aligned}$$

where the Fermi velocity $v_F=2t\sin k_F$. The $g_{1\perp }$ term describes backscattering processes, regardless of the electron fillings. Significantly differently, the $g_{3\perp }$ terms is the umklapp process, which is active at $\nu =1/2$. The origin of phase factors $e^{i(\frac{1}{2}-\nu )Gx}$ is shown in the Appendix, which explicitly shows that in addition to the half filling ($\nu =1$), the umklapp processes take place at quarter filling ($\nu =1/2$) in the model involved.

According to bosonization scheme, the fermion operators are expressed by bosonic operators $\phi _{r\alpha }(x)$^28^

6 $$\begin{aligned} \psi _{r\alpha }(x)=\frac{U_{r\alpha }}{\sqrt{2\pi \epsilon }}e^{i\phi _{r\alpha }(x)}, \end{aligned}$$

with

7 $$\begin{aligned} \phi _{r\alpha }(x)=\pm 2\pi \int \rho _{r\alpha }(x)\mathrm {d}x, \end{aligned}$$

where the upper (lower) sign corresponds to $r=L (R)$. $\epsilon$ is a short-distance cutoff. The Hermitian operators $U_{r\alpha }$ ensure anticommutation relations of different fermion fields^29^. In terms of $\phi _{r\alpha }(x)$, we define

8 $$\begin{aligned} \phi _{r\rho }(x)&=  {} \frac{\phi _{r\uparrow }(x)+\phi _{r\downarrow }(x)}{\sqrt{2}},\nonumber \\ \phi _{r\sigma }(x)&=  {} \frac{\phi _{r\uparrow }(x)-\phi _{r\downarrow }(x)}{\sqrt{2}}. \end{aligned}$$

Based on this, we further introduce a pair of canonical variables $\phi _\mu (x)$ and $\theta _\mu (x)$,

9 $$\begin{aligned} \phi _\mu (x)&=  {} \frac{\phi _{L\mu }+\phi _{R\mu }}{\sqrt{4\pi }}=\sqrt{\pi }\int \left( \mu _L+\mu _R\right) \mathrm {d}x, \end{aligned}$$

10 $$\begin{aligned} \theta _\mu (x)&=  {} \sqrt{\pi }\int \left( \mu _R-\mu _L\right) \mathrm {d}x, \end{aligned}$$

which describe the charge ($\mu =\rho$) and spin ($\mu =\sigma$) fields, respectively, satisfying the relation

11 $$\begin{aligned} \left[ \phi _\mu (x), \theta _{\mu ^\prime }\left( x^\prime \right) \right] =\frac{\pi }{2}\delta _{\mu \mu ^\prime }sgn\left( x-x^\prime \right) . \end{aligned}$$

In the present work, we focus only on the quarter-filling case, at which the umklapp process is absent in the 1D standard Hubbard model. Using the expressions (6)–(11), we convert the Hamiltonian (5) into the sum of the charge and spin sectors, $H=H_\rho +H_\sigma$, with

12 $$\begin{aligned} H_\rho&=  {} \frac{v_F}{2}\int \mathrm {d}x\left[ \left( 1-\frac{g_\rho }{2}\right) \left( \partial _x\phi _\rho \right) ^2 +\left( 1+\frac{g_\rho }{2}\right) \left( \partial _x\theta _\rho \right) ^2\right] \nonumber \\&+\frac{v_Fg_{3\perp }}{2\pi \epsilon ^2}\int \mathrm {d}x\cos \left( \sqrt{8\pi }\phi _\rho \right) , \end{aligned}$$

13 $$\begin{aligned} H_\sigma &=  {} \frac{v_F}{2}\int \mathrm {d}x\left[ \left( 1-\frac{g_\sigma }{2}\right) \left( \partial _x\phi _\sigma \right) ^2 +\left( 1+\frac{g_\sigma }{2}\right) \left( \partial _x\theta _\sigma \right) ^2\right] \nonumber \\&+\frac{v_Fg_{1\perp }}{2\pi \epsilon ^2}\int \mathrm {d}x\cos \left( \sqrt{8\pi }\phi _\sigma \right) . \end{aligned}$$

Up to the lowest orders of interactions, the dimensionless coupling constants are calculated as

14 $$\begin{aligned} g_{\rho } &=  {} -\frac{U_e+U_o}{2\pi v_F}, \end{aligned}$$

15 $$\begin{aligned} g_{3\perp } &=  {} \frac{U_o-U_e}{2\pi v_F}, \end{aligned}$$

16 $$\begin{aligned} g_\sigma &=  {} g_{1\perp }=\frac{1}{2\pi v_F}\left( U_e+U_o-2J\right) . \end{aligned}$$

In the above expressions, the relation $g_\sigma =g_{1\perp }$ comes directly from the spin SU(2) symmetry of the model.

## Renormalization group analysis

In addition to free terms, the bosonized Hamiltonian contains in general interacting terms, which are signed by the cosines in Eqs. (12) and (13). Their effects can be analyzed by the renormalization-group (RG) method. To be specific, if the coefficient of a cosine decreases in the RG flow, the fixed point corresponds to a trivial theory of free bosons with known properties. On the contrary, if the RG flow scales towards strong-coupling regions, the coefficient increases. At this point, the fields are trapped in a minimum of the free energy and the different states can be characterized by computing the vacuum expectation value at this minimum of the bosonic operators^30^. To perform the RG scheme, we define a nonuniversal parameter closely related to both fixed-point values and correlation functions as

17 $$\begin{aligned} K_\mu =\sqrt{\frac{2+g_\mu }{2-g_\mu }}, \quad (\mu =\rho ,\sigma ). \end{aligned}$$

In the weak-coupling case, one has $|g_\mu |,|g_{i\perp }|\ll 1$, and the general relation (17) implies

18 $$\begin{aligned} K_\mu &=  {} 1+\frac{g_\mu }{2}+\mathcal {O}\left( g_\rho ^2\right) +\cdots \simeq 1+\frac{g_\mu }{2}, \end{aligned}$$

19 $$\begin{aligned} \frac{1}{K_\mu } &=  {} 1-\frac{g_\mu }{2}+\mathcal {O}\left( g_\rho ^2\right) -\cdots \simeq 1-\frac{g_\mu }{2}. \end{aligned}$$

In terms of the expressions (17)–(19), the low-energy behavior of the models (12) and (13) can be descried by the known sine-Gordon models for the charge and the spin sectors,

20 $$\begin{aligned} H_\mu &=  {} \frac{v_\mu }{2}\int \left[ K_\mu ^{-1}\left( \partial _x\phi _\mu \right) ^2+K_\mu \left( \partial _x\theta _\mu \right) ^2\right] \mathrm {d}x \nonumber \\&+\frac{v_\mu m_\mu }{2\pi \epsilon ^2}\int \cos \left( \sqrt{8\pi }\phi _\mu \right) \mathrm {d}x. \end{aligned}$$

Here $v_\rho m_\rho =v_Fg_{3\perp }$, $v_\sigma m_\sigma =v_Fg_{1\perp }$. The velocities of charge ($\rho$) and spin ($\sigma$) excitations become modified as $v_\mu =v_F/K_\mu$. The effects of umklapp and backward processes are thus associated with scaling behavior of the effective masses ($m_\mu$), which are accomplished by the RG analysis. For this purpose, we introduce the following RG equations^31^

21 $$\begin{aligned}&\frac{\mathrm {d}\xi _{\mu }(l)}{\mathrm {d}l}=-\eta ^2_{\mu }(l), \end{aligned}$$

22 $$\begin{aligned}&\frac{\mathrm {d}\eta _{\mu }(l)}{\mathrm {d}l}=-\xi _{\mu }(l)\eta _{\mu }(l), \end{aligned}$$

with the initial conditions $\xi _{\mu }(0)=4(\sqrt{K_\mu }-1)\simeq g_\mu$, $\eta _{\rho }(0)=m_\rho \simeq g_{3\perp }$, and $\eta _{\sigma }(0)=m_\sigma \simeq g_{1\perp }$.

The RG flows, which are characterized by scaling invariant $\xi _{\mu }^2-\eta _{\mu }^2$=const, exhibits that the system is gapless (gapped) if the bare parameters $\xi _\mu (0)\ge |\eta _{\mu }(0)|$ ($\xi _\mu (0)<|\eta _{\mu }(0)|$ ). In the gapless case, the field $\phi _\mu (x)$ oscillates, and the integral of the cosine in Eq. (20) vanishes averagely. With increasing scaling length, the effective mass $m_\mu (l)\rightarrow 0$, and the low energy behavior of the system is described by a free scalar field. In the gapped case, $m_\mu (l)\rightarrow \pm \infty$, and the vacuum expectation value of the field $\langle \phi _\mu \rangle$ is pinned to a value that minimizes the potential. Depending on the sign of the initial mass, $\langle \phi _\mu \rangle =\sqrt{\pi /8}$ for $m_\mu >0$, and $\langle \phi _\mu \rangle =0$ for $m_\mu <0$^16^.

In the spin sector, the SU(2) symmetry drives the RG flux flow only along the separatrix $\xi _\sigma =\eta _{\sigma }$. This causes the spin-gap transition to takes place at $g_\sigma =g_{1\perp }=0$, which is equivalent to the condition

23 $$\begin{aligned} U_o+U_e=2J. \end{aligned}$$

For $U_o+U_e\ge 2J$, the spin excitation is gapless, $\Delta _\sigma =0$. The backward processes are irrelevant, and the correlation exponent $K_\sigma (l\rightarrow \infty )=K_\sigma ^*=1$. In the opposite region, the excitation spectrum is gapped, $\Delta _\sigma >0$. The backscattering processes are relevant, $K_\sigma ^*=0$, and $\langle \phi _\sigma \rangle =0$.

As not in the spin sector, the SU(2) symmetry is broken in the charge sector. Furthermore, the parameters $K_\rho$ and $m_\rho$ vary independently, and the RG flux deviates from the separatrix $\xi _\rho (l)=\eta _{\rho }(l)$. Since $\xi _\rho (0)<0$ and $\eta _\rho (0)\ne 0$, the charge excitation is gapped. This indicates that the umklapp processes are always relevant. There is no charge-gap transition, instead, a Gaussian transition takes place at $\eta _\rho (0)=0$ and $\xi _\rho (0)<0$. This is a type of transition between the two massive phases, which correspond to the opposite fixed points, $\eta _\rho (l\rightarrow \infty )=\pm \infty$, and the charge gap only closes at a critical point. According to Eq. (15), the Gaussian transition point is obtained as

24 $$\begin{aligned} U_o=U_e. \end{aligned}$$

The value of $\langle \phi _\rho \rangle$ is $\sqrt{\pi /8}$ for $U_o>U_e$ and zero for $U_o<U_e$, respectively. In the two cases, we have $K_\rho ^*=0$.

## Ground-state phase diagram

In this sector, we determine ground-state phase diagram of the system. Because the umklapp processes are always present, the system is an insulator with a charge gap ($\Delta _\rho >0$). In one dimension, the insulating phases are usually described by several types of density waves. For this purpose, we need introduce a set of order parameters. Given the non-half filling case, it is only necessary to use site-located $\mathcal {O}_{SDW}$ and $\mathcal {O}_{CDW}$, which describe the SDW and CDW instabilities, respectively. The bond-located orderings $\mathcal {O}_{BSDW}$ and $\mathcal {O}_{BCDW}$ do not need to be introduced, because they are a special feature of the half-filled band case [23]. At quarter filling, $k_F=\pi /4$, and we have

25 $$\begin{aligned} \mathcal {O}_{SDW} &=  {} e^{i\pi j/2 }\left( n_{j\uparrow }-n_{j\downarrow }\right) \sim \cos \left( \frac{\pi }{2}j-\sqrt{2\pi }\phi _\rho \right) \sin \sqrt{2\pi }\phi _\sigma , \end{aligned}$$

26 $$\begin{aligned} \mathcal {O}_{CDW} &=  {} e^{i\pi j/2 }\left( n_{j\uparrow }+n_{j\downarrow }\right) \sim \sin \left( \frac{\pi }{2}j-\sqrt{2\pi }\phi _\rho \right) \cos \sqrt{2\pi }\phi _\sigma . \end{aligned}$$

At odd and even sites, they are explicitly written as

$$\begin{aligned} \mathcal {O}_{SDW} &=  {} \left\{ \begin{array}{ll} \mathcal {O}_{SDW}^{(1)}\sim \cos \sqrt{2\pi }\phi _\rho \sin \sqrt{2\pi }\phi _\sigma &{}{(j\in even)}\\ \mathcal {O}_{SDW}^{(2)}\sim \sin \sqrt{2\pi }\phi _\rho \sin \sqrt{2\pi }\phi _\sigma &{}{(j\in odd),} \end{array} \right. \\ \mathcal {O}_{CDW}& =  {} \left\{ \begin{array}{ll} \mathcal {O}_{CDW}^{(1)}\sim \sin \sqrt{2\pi }\phi _\rho \cos \sqrt{2\pi }\phi _\sigma &{}{(j\in even)}\\ \mathcal {O}_{CDW}^{(2)}\sim \cos \sqrt{2\pi }\phi _\rho \cos \sqrt{2\pi }\phi _\sigma &{}{(j\in odd).} \end{array} \right. \end{aligned}$$

**Figure 1:**
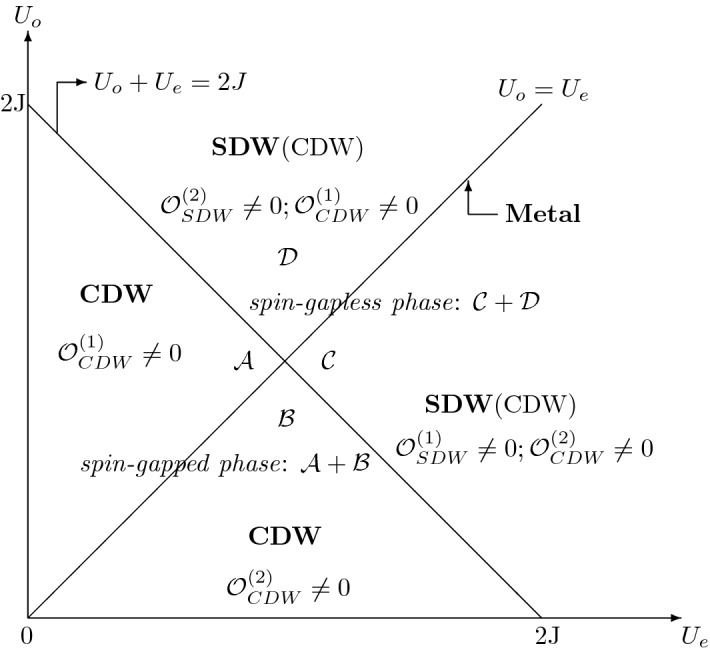
The weak-coupling phase diagram of 1D unusual *t*–*U*–*J* model with alternating on-site repulsion at quarter filling. The subleading instability is indicated in bracket.

The ground state phase diagram is shown in Fig. 1, in which the transition lines (23) and (24) divide the ($U_o, U_e$) plane into four regions.

Region $\mathcal {A}$: $U_e<U_o<2J-U_e$. The initial couplings are $g_{3\perp }>0$ and $g_{1\perp }<0$, so the expectation values are ordered with $\langle \phi _\rho \rangle =\sqrt{\pi /8}$ and $\langle \phi _\sigma \rangle =0$. The fixed points correspond to $g_{3\perp }^*=+\infty$ and $g_{1\perp }^*=-\infty$. Except for $\mathcal {O}_{CDW}^{(1)}$, the other order parameters take zero. In the region $\mathcal {B}$: $U_o<\min \{U_e; 2J-U_e\}$. As in the region $\mathcal {A}$, the backward and umklapp processes are both relevant, while the negative initial couplings lead to $g_{3\perp }^*=g_{1\perp }^*=-\infty$, accompanied by $\langle \phi _\rho \rangle =\langle \phi _\sigma \rangle =0$. Apparently, only the order parameter $\mathcal {O}_{CDW}^{(2)}$ takes nonzero. Consequently, the system is an insulator with fully gapped CDW instability in the regions of both $\mathcal {A}$ and $\mathcal {B}$.

Region $\mathcal {C}$: $2J-U_e<U_0<U_e$. The backward term is marginally irrelevant, with the fixed point $g_{1\perp }^*=0$, and the spin sector is gapless, with the correlation exponent $K_\sigma ^*=1$. In the gapped charge sector, $g_{3\perp }^*=-\infty$, and $\langle \phi _\rho \rangle =0$. At the same time, the spin field $\phi _s$ is fluctuating, not to be locked at a fixed value. The values of both $\mathcal {O}_{CDW}^{(1)}$ and $\mathcal {O}_{SDW}^{(2)}$ are equal to zero, however, $\mathcal {O}_{SDW}^{(1)}$ and $\mathcal {O}_{CDW}^{(2)}$ take nonzero values. In the region $\mathcal {D}$: $U_o>\max \{U_e; 2J-U_e\}$. Depending on the RG analysis, we obtain $g_{3\perp }^*=+\infty$, $g_{1\perp }^*=0$, and $\langle \phi _\rho \rangle =\sqrt{\pi /8}$. As a result, $\mathcal {O}_{SDW}^{(1)}=\mathcal {O}_{CDW}^{(2)}=0$. On the contrary, $\mathcal {O}_{CDW}^{(1)}$ and $\mathcal {O}_{SDW}^{(2)}$ take nonzero values. To further determine dominant instabilities, we need compute the corresponding correlation function (*C*), which is defined at a large distance as $C_i(x)=\langle \mathcal {O}_i(x)\mathcal {O}^\dag _i(0)\rangle$^20^. In these two regions, we have

27 $$\begin{aligned}C_{SDW}(x)\propto x^{-1}\ln ^{1/2} x, \end{aligned}$$

28 $$\begin{aligned}C_{CDW}(x)\propto x^{-1}\ln ^{-3/2} x. \end{aligned}$$

It is easily observed that the correlation of the CDW type decays faster than that of the SDW one at large distances, but for any distance both are nonzero. Therefore, the SDW and CDW instabilities coexist in the $\mathcal {C}$ and $\mathcal {D}$ regions. However, due to the weakly logarithmical corrections, the SDW correlation dominates over the CDW one in the spin-gapless insulating phase. In fact, the coexisting SDW and CDW phenomenon has been extensively studied in organic charge-transfer solid^33^.

As expected, on the boundary $U_o=U_e$, the conventional *t*–*U*–*J* model is recovered. In this case, the umklapp processes disappear. Because of massless charge excitations, the system demonstrates metallic behavior.

## Summary and discussion

To investigate the occurrence of umklapp processes away from half filling, we have studied a unusual 1D extended Hubbard model, which, in addition to the conventional inter-site Heisenberg exchange interaction, includes alternating on-site repulsive interactions on odd and even lattice sites at quarter filling. In order to analyze the model analytically, we focus on the weak-coupling case, where the bosonization and renormalization group techniques are effectively applied to the 1D system. We find that at quarter filling the unequal Hubbard interactions lead to the generation of umklapp processes, and the charge excitations are always gapped. This behavior is completely different from that of the usual $U_o=U_e$ case, where the umklapp processes are absent, characterized by the gapless charge excitations. As usual, the backward processes are not affected, and the spin sector is either gapped or gapless, based on the relation of $U_o$, $U_e$ and *J*. The spin excitation is just as in the usual extended Hubbard chain with the spin-SU(2) symmetry. The spin-gap transition divides the phase diagram into two different insulating phases. For $U_e+U_o<2J$, the system is in a spin-gapped phase with the CDW instability; for $U_e+U_o\ge 2J$, the system is in a spin-gapless phase, characterized by the coexistence of CDW and SDW instabilities, but the SDW correlation dominates over the CDW one.

Moreover, the SDW and CDW states are independent of values of $U_a$ and $U_b$. According to the lattice-translation invariance of the system, the Hamiltonian is unchanged when we shift one lattice, but the $U_o$ and $U_e$ interactions interchange each other. Physically, using the periodic boundary condition, the ground state for $U_o>U_e$ and $U_o<U_e$ should be the same. This is clearly seen from the phase diagram, where only two phase are present, and they are separated by the spin-gap transition line $U_e+U_o=2J$, which is determined by the competition between on-site interaction and spin exchange interaction. Equivalently, the phase diagram is not modified for the difference between $U_e$ and $U_o$, including any large $U_e-U_o$.

Note that our result depends on 1D calculations, and it cannot be generalized to the 2D case, e.g., high-$T_c$ cuprates, although unequal on-site interactions do exist there. This is because in two dimensions the umklapp processes are practically regardless of the filling provided $|k_F|$ is large enough, as is completely different from the 1D case. Furthermore, the 2D system include more complicated on-site and spin exchange interactions. Maybe, the result can be applied to the materials containing edge-sharing Cu-O chains, where the low-energy physics is one-dimensional.
